# Driven by notifications – exploring the effects of badge notifications on user experience

**DOI:** 10.1371/journal.pone.0270888

**Published:** 2022-06-30

**Authors:** Nicola Bartoli, Simone Benedetto

**Affiliations:** Jakala Experience Lab, JAKALA S.p.A. S.B. s.u., Milan, Italy; National Textile University, PAKISTAN

## Abstract

Notification badges are an unexplored category of visual feedback to which we are continuously exposed. This study aims to deepen knowledge on the topic by measuring the behavioral effects of notification badges on a large sample of smartphone users. More precisely, the goal of the study is to observe if the presence of notification badges increases the frequency of clicks on apps. More than 1000 participants were involved in a remote between-subjects experiment, allocated into fifteen equinumerous groups of comparison. Each participant was presented with a smartphone screen displaying fifteen app icons and just one badge notification. Participants were asked to perform a remote user test called First Impression Click Test: a methodology that indicates where they would click first to accomplish a given task (i.e., *Where would you click first on this screen*?). Our results show a large increase in the number of clicks on apps with notification badges compared to those without notification badges and suggest the important ability of these small affordances to attract attention and stimulate action. Based on the evidence provided, our findings have practical implications for user experience design.

## Introduction

In recent years we have witnessed an exceptional growth in the spread of smartphones and in the use of applications. The number of smartphone subscriptions worldwide today surpasses six billion and is forecasted to further grow by several hundred million in the next few years (Statista, 2021; www.statista.com/topics/840/smartphones). On average, smartphone owners check their phone every 7 minutes, use 9 to 10 different apps per day and about 30 apps each month (App Annie–The state of mobile, 2021; www.appannie.com/en/go/state-of-mobile-2021). Applications like messaging, e-mail, and online social networks allow smartphone users to connect with their family, friends and even co-workers wherever they are. Most of these apps use proactive, push-based notifications (i.e., visual, auditory, and/or haptic alerts) to inform smartphone users about new, unattended messages or events, even when they are not actively using their device. Smartphones offer various methods for the user to receive notifications, including a status bar, LED indicator, pop-up screen, badge, vibration, sound, or a combination of these [1]. In this context, a badge is the red circle that appears on the upper right-hand corner of the app’s icon, capable of capturing and holding user attention. The white number inside the red circle represents the number of unread notifications waiting to be opened.

By visually showcasing notifications that a user hasn’t yet engaged with, badge notifications can trigger two main cognitive biases: salience and sense of urgency. Salience bias (also known as perceptual salience) is the cognitive bias that predisposes individuals to focus on items that are more visually prominent or emotionally striking and ignore those that are unremarkable, even though this difference is often irrelevant by objective standards. This creates a bias in favor of things that are striking and perceptible [2–4]. Sense of urgency is a cognitive bias or a systematic error of thinking, that puts people into reactive mode and pushes them to choose one option rather than another based on which they perceive most urgent, even if that choice is objectively worse or it distracts from long-term goals. In other words, people are psychologically inclined to put off meaningful work in favor of tasks that feel more urgent [5, 6]. Notification badges can therefore be considered as wordless but meaningful calls to action, triggering those cognitive biases with minimal effort.

While previous studies have focused on notifications in the broadest sense of the term, very little is known about smartphone badge notifications and their impact on a user’s behavior.

Several works have focused on the effects of desktop [7, 8] and mobile notifications [9–17]. Fallman & Yttergren [9] proposed a system that adapts smartphone notifications according to situational circumstances, Garzonis et al. [10] studied intuitiveness, learnability, memorability, and user preference of two types of audio notifications on the smartphone. Iqbal & Horvitz [7] and Mark, Voida and Cardello [8] investigated the role of desktop notifications in work environments and highlighted both their interruptive character and their stressful effect on those who receive them while carrying out a primary task. Fischer et al. [12], on the other hand, conducted a study on the timing of mobile notifications, finding that they are processed faster if they arrive at the end of an interaction with their device (call or SMS). Saket et al. [13] observed the urgency effect generated by ten different vibration modes as a notification system on smartphones. Pielot et al. [14] worked on finding predictors of the time it takes a user to interact with the notification of a mobile message.

One of the latest works published compared the effectiveness of two types of notification (push vs badge) in informing software developers about the availability of software updates and found that, on average, systems that use badge notifications are slightly less effective than those based on push notifications [18]. Morrison et al. [15] published an exploratory study on the potential impact of time (intelligent and non-intelligent) and the frequency (daily and occasional) of responding to push notifications on the use of a stress management tool on smartphones. The authors found that frequent notifications can encourage greater exposure to intervention content without discouraging engagement, but adaptive tailoring of notification timing does not always enhance their use. Fitz et al. [16] tested whether delivering notifications in predictable intervals throughout the day could improve psychological well-being and found that those who received notifications three times a day felt more attentive, productive, in a better mood, and more in control of their phones. with respect to those who received notifications in unpredictable ways. Loid, Täht and Rozgonjuk [17] found that notifications regarding excessive smartphone use did not lower self-reported scores about problematic smartphone use, nor participants’ screen time or the frequency of phone-checking behaviour.

With the aim of filling the literature gap and shedding light on smartphone badge notifications and their impact on user behavior, we conducted a remote and unmoderated test called First Impression Click Test, which allows to collect data on where, on an interface, people would click first to complete a given task [19, 20].

The goal of the study was to observe if the presence of badges would have increased the frequency of clicks on apps. To this end more than 1000 participants were involved in a remote between-subjects experiment, allocated into fifteen equinumerous groups of comparison. Each participant was presented with a smartphone screen displaying fifteen app icons and just one badge notification. Every screen was identical to the other, except for the app icon on which the badge notification was placed.

## Materials and methods

### Participants

We recruited 1095 participants (55% women, mean age = 38 years, SD = 10) through an online panel to take part in the experiment. All of them self-declared as smartphone users but were naïve as to the aims and the expected outcomes of the experiment. The experimental group was allocated to 15 equinumerous groups of comparison (73 participants each). We eventually rejected 86 participants out of 1095 from all analyses because of poor data quality (i.e. participants clicking on regions other than icons). A small financial compensation was offered to participants. The study was performed in keeping with the Declaration of Helsinki. All participants gave written informed consent before participation. The JAKALA Experience Lab Ethics Committee approved the study.

**Fig 1:**
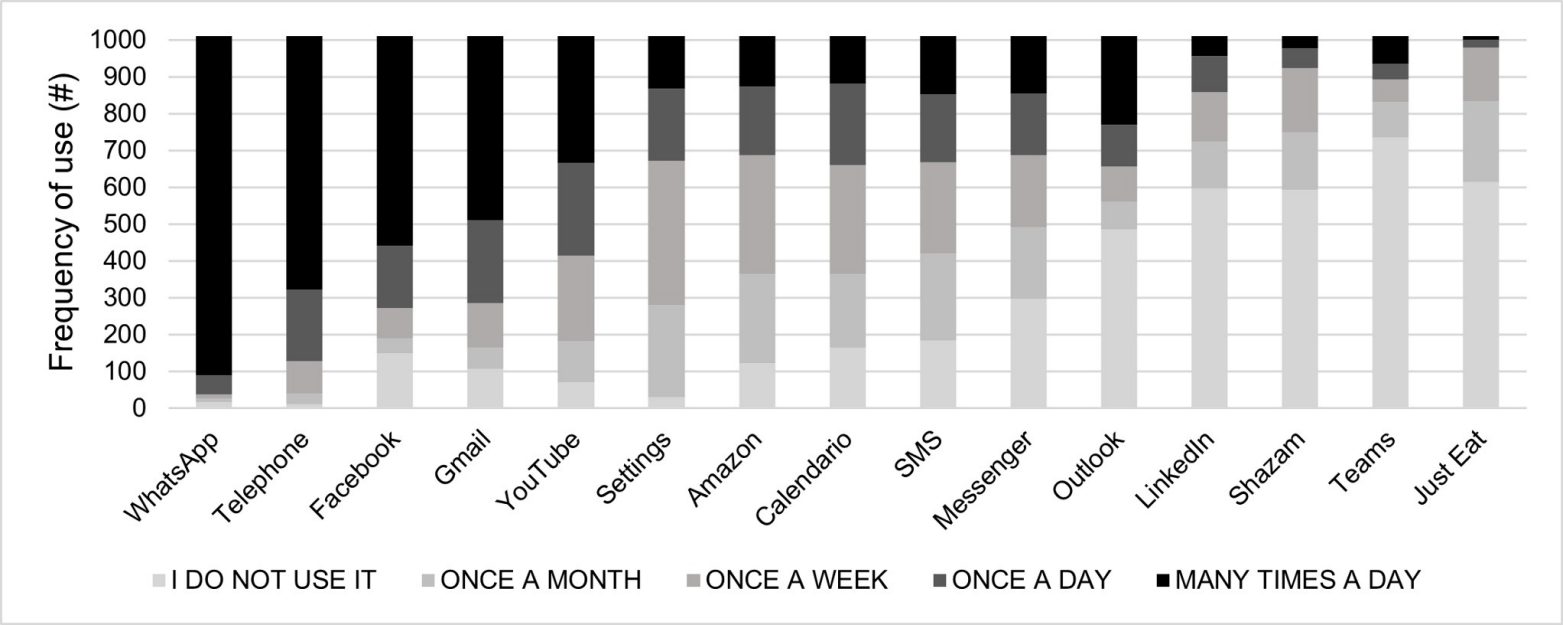
Apps usage habits (N = 1009). Results have been displayed according to the frequency of use (descending order).

At the end of the experimental session, participants completed a short questionnaire regarding their app’s ordinary usage habits (Fig 1). The questionnaire investigated the frequency of use of the 15 apps shown during the test using a 5-point Likert scale (from 1 = "I never use it" to 5 = "I use it several times a day"). Participants declared that the apps they use the most were WhatsApp, Telephone Facebook, and Gmail while the ones they use the least were LinkedIn, Shazam, Teams and Just Eat.

### Apparatus and stimuli

The First Impression Click Test is a user research methodology that allows one to collect first impressions regarding static interfaces and more specifically where people will click first to accomplish a given task [20]. This remote test is often used to validate templates and design choices, but additionally, it allows one to investigate the elements of an interface that most attract users’ attention and to make inferences on their information search patterns. In our study, the First Impression Click Test was carried out remotely through a dedicated online platform (Optimal Workshop-Chalkmark; www.optimalworkshop.com/chalkmark).

Each of the fifteen groups of participants (15 groups x 73 participants) carried out the remote test performing just one task on a single home screen of an Android smartphone with one notification badge placed on just one app out of the fifteen in total. The given task required participants to freely select the area that they would have clicked on first when viewing the screen. The task was thus formulated: “*Where would you click first on this screen*?”. The test ended when the participants clicked on the desired element of the interface. At the end of the test, a short questionnaire on apps use habits was administered.

The apps chosen for the home screen of an Android smartphone were selected according to their total number of downloads and frequency of use (App Annie–The State of Mobile 2021 Report; www.appannie.com/en/go/state-of-mobile-2021), aiming to reproduce a realistic and representative set of apps for Italian users. We preferred to employ Android in lieu of iOS since the former represents about the 75% of market share and is therefore the most common device among the Italian population (Statista, 2022; www.statista.com/statistics/623153/leading-mobile-operating-systems-ranked-by-market-share-in-italy). In addition to the apps available by default on a smartphone (Calendar, Telephone, SMS, Settings), nine further apps were selected:

- social media (Facebook, LinkedIn);
- messaging and email (WhatsApp, Messenger, Teams, Gmail and Outlook);
- shopping (Amazon, Just Eat);
- entertainment (YouTube, Shazam).

### Experimental design and procedure

A between-subject design was employed. Participants were evenly distributed among 15 groups of the same size. Each of the fifteen groups of participants was exposed to a single experimental condition (one smartphone interface with the notification badge on a single app). Each experimental session lasted 90 seconds on average and consisted of:

1. reading a short instructions page for the first impression click test;
2. performing the first impression click test;
3. filling in a questionnaire on apps use habits and sociodemographic profile.

### Dependent variable

To test the hypothesis, the percentage of clicks on the apps was employed as the only dependent variable. The given task consisted, for each of the participants, of just one single click on the chosen app. Every participant could therefore select only 1 app out of 15 for each experimental session. Therefore, the percentage of clicks was a dichotomous variable which contained two distinct values: 100% and 0%. The first value (100%) was attributed to 1 single app out of 15, the second (0%) to the rest of the apps (14 out of 15).

## Results

The significance level α was set at .05 for all statistical analyses. The percentage of clicks was analyzed with a Kruskal–Wallis H test, a non-parametric method for comparing two or more independent samples of equal or different sample sizes. The adjusted Mann–Whitney U test [21, 22] was instead used for planned comparisons (Badge vs. No badge). Means, standard deviations and sample sizes for each of the dependent variables and experimental conditions are reported in Table 1.

**Table 1:** Means, standard deviations and sample sizes for each of the experimental conditions.

	Badge			No badge		
	%	SD	Size(n)	%	SD	Size(n)
WhatsApp	79,7	40,6	64	38,2	48,6	945
Teams	18,5	39,1	68	0,6	7,9	941
Messenger	20,7	40,9	58	1,6	12,5	951
Just Eat	12,1	32,9	66	0,7	8,6	943
Amazon	39,1	49,2	64	8,5	27,9	945
Facebook	44,1	50	68	10	30	941
LinkedIn	16,7	37,5	72	0,9	9,2	937
Calendar	17,7	38,5	62	0,8	9,2	947
Outlook	16,4	37,3	73	1,4	11,7	936
Gmail	21,5	41,4	65	6,8	25,2	944
YouTube	23,9	43	71	7,9	27	938
Shazam	9,7	29,8	72	0,3	5,7	937
Telephone	26,8	44,6	71	1,4	11,7	938
SMS	17,9	38,6	67	0,3	5,6	942
Settings	10,3	30,6	68	1,2	10,8	941

**Fig 2:**
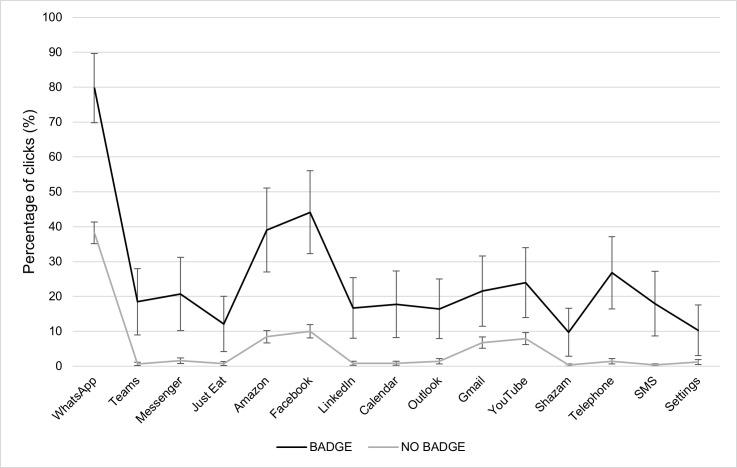
Percentage of clicks for each of the apps and experimental conditions (badge vs. no badge) with relative confidence intervals.

To test the hypothesis that the presence of a badge increases the percentage of clicks on that app, each of the apps with the badge was compared (one at a time) to the group constituted by the rest of the apps with no badge (n = 14). Results showed that the presence of the notification badge systematically captures more clicks with respect to the condition in which the badge is unavailable. As to the interactions with gender and age no results emerged between the two groups (i.e Badge vs. No badge). Main effects and planned contrasts for each of the apps are shown in Table 2. Results are reported in Fig 2.

**Table 2:** Main effects and planned contrasts for each of the apps. Main effects were calculated using the Kruskal–Wallis H test (all ps < .001). Planned contrasts were calculated with the adjusted Mann–Whitney U test (all ps < .001).

	Main Effect	Planned contrast (Badge vs. No badge)
WhatsApp	H (14, N = 1009) = 64,07	Z = 6,53
Teams	H (14, N = 1009) = 118,17	Z = 10,49
Messenger	H (14, N = 1009) = 82,97	Z = 8,75
Just Eat	H (14, N = 1009) = 60,43	Z = 7,38
Amazon	H (14, N = 1009) = 70,77	Z = 7,75
Facebook	H (14, N = 1009) = 84,73	Z = 8,27
LinkedIn	H (14, N = 1009) = 94,43	Z = 9,27
Calendar	H (14, N = 1009) = 110,75	Z = 9,47
Outlook	H (14, N = 1009) = 64,14	Z = 7,96
Gmail	H (14, N = 1009) = 35,91	Z = 4,30
YouTube	H (14, N = 1009) = 29,97	Z = 4,55
Shazam	H (14, N = 1009) = 66,86	Z = 7,75
Telephone	H (14, N = 1009) = 143,22	Z = 11,75
SMS	H (14, N = 1009) = 132,31	Z = 11,39
Settings	H (14, N = 1009) = 39,69	Z = 5,48

## Discussion

The goal of the present study is to investigate the impact of badge notifications on smartphone users’ behavior. Regardless of the app type, the presence of the notification badge systematically captured more clicks with respect to the condition in which the badge is unavailable. Based on the evidence provided, our findings highlight the strong impact of badge notifications and have practical implication for user experience design.

These results can be explained by to two main theories that refer to cognitive biases. Cognitive biases are systematic patterns of deviation from norm or rationality in judgment [23]. They arise from erroneous perceptions, from which judgments, prejudices and ideologies are inferred because of the brain’s attempt to simplify information processing [2, 3, 24]. In everyday life, cognitive biases can be beneficial because they do not require much mental effort and they allow people to make decisions quickly without subjecting each decision to careful criticism or judgment which takes time and energy. Cognitive biases are activated by what Kahneman defines as System 1, the most primordial component of our brain to which the automatic and fast processes belong (e.g., perception and intuition) and which prevails when faced with apparently simple problems or which suggest an immediate answer [25]. The solutions provided by System 1 are sometimes accepted beyond conscious and rational judgment and intuitions or perceptions, even if incorrect, can turn into behaviors: in this context, the slower one relating to logical processes such as rational thinking and reasoning (i.e., System 2), is not activated.

Among the various theories that have studied cognitive biases there are two that allow us to interpret the results of this study: the first refers to the saliency bias [4] and the second to the urgency bias [5, 6]. On the one hand, the salience bias describes the human tendency to focus on items or information that are more noteworthy while ignoring those that do not grab our attention. In our case, the notification badges, due to their physical characteristics (e.g., color, shape, positioning), create a salience bias in favor of those apps that, from a visual and perceptive level, instantly become more relevant than the others. On the other hand, the urgency bias describes the tendency for people to perform unimportant tasks over important tasks when the unimportant tasks are characterized merely by spurious urgency (and illusion of expiration). In this respect, the notification badges produce a reactive effect on a cognitive-emotional level and push people, due to the implicit meaning they convey, to choose an app rather than another based on the one they perceive as most urgent and important.

The results of our study can be considered pioneering in the field of notification badge design as there is no evidence in the literature regarding the topic of the study. Future studies might investigate the demographic characteristics of the participant sample or the physical aspects of the notification badges. As to the sample, participants from different nationalities and with different app usage habits might be involved. As to the manipulation of factors related to the notification badge, it might be useful to include factors such as the number shown in the badge (digit other than 1), the color and the position. Also, it might be interesting to compare badge notifications with other types of smartphone notifications such as status bar, LED indicator, pop-up screen, vibration, sound, or a combination of these. This will allow us to broaden the knowledge of the effect of these messages on people’s behavior and the awareness of the stimuli to which we are exposed every day.

## Conclusions

Notification badges represent a category of warning to which we are continuously exposed, and which needs to be investigated since there is little knowledge in the literature regarding their effects on human behavior. This study aims to deepen the knowledge on the topic by measuring the effects of notification badges on the behavior employing a large sample of smartphone users. Our results show a large increase in the number of clicks on apps with notification badges compared to those without notification badges and suggest the important ability of these small affordances to attract attention and stimulate action.

## Supporting information

S1 Dataset Study data. (XLSX)
